# Coupling of Ammonium Dihydrogen Phosphate Additives with LiPF_6_ Electrolytes for Improving Thermal Stability and Performance of Lithium-Ion Batteries

**DOI:** 10.3390/mi16090966

**Published:** 2025-08-22

**Authors:** M. Thien Phung, T. Thu Phuong Vu, Seung Beop Lee, Ing Kong, Min Kim, Mohammad Shaheer Akhtar, O-Bong Yang

**Affiliations:** 1 Graduate School of Integrated Energy-AI, Jeonbuk National University, Jeonju 54896, Republic of Koreaobyang@jbnu.ac.kr (O.-B.Y.); 2 School of International Engineering and Science, Graduate School of Integrated Energy-AI, Jeonbuk National University, Jeonju 54896, Republic of Korea; 3 Advanced Polymer and Composite Materials Laboratory, Department of Engineering, School of Computing, Engineering and Mathematical Sciences, La Trobe University, Bendigo, VIC 3552, Australia; i.kong@latrobe.edu.au; 4 School of Semiconductor and Chemical Engineering, Clean Energy Research Center, Jeonbuk National University, Jeonju 54896, Republic of Korea; 5 New and Renewable Energy Materials Development Center (NewREC), Jeonbuk National University, Jeonju 54896, Republic of Korea; 6 Department of Industry-Academia Convergence Research, JBNU-KIST, Jeonbuk National University, Jeonju 54896, Republic of Korea

**Keywords:** liquid electrolyte, ammonium dihydrogen phosphate, additive, thermal stability, lithium-ion battery

## Abstract

The flammability and volatility of conventional lithium hexafluorophosphate (LiPF_6_)-based electrolytes with organic carbonate solvents remain critical issues to the safety and thermal stability of lithium-ion batteries (LIBs). This study investigates the incorporation of phosphate-based additives including ammonium dihydrogen phosphate (ADP), trimethyl phosphate (TMP), and trimethyl phosphite (TMPi) into LiPF_6_ electrolytes for improving the ionic conductivity, safety, and electrochemical performance of LIBs. Self-extinguishing time (SET) measurements demonstrated that the ADP-based LiPF_6_ electrolyte significantly reduced flammability, achieving a shorter SET of 04 min 53 s, compared to 12 min for the pristine LiPF_6_ electrolyte. The ADP-based LiPF_6_ electrolyte possessed the highest ionic conductivity (14.08 mS·cm^−1^) with an excellent lithium-ion transference number of 0.0076. Electrochemical impedance spectroscopy (EIS) and cyclic voltammetry (C-V) analyses demonstrated that ADP lowered interfacial resistance and stabilized long-term cycling behavior. In particular, the 1% ADP-based LiPF_6_ electrolyte maintained improved charge-discharge profiles and Coulombic efficiency over 200 cycles. These results highlight ADP’s dual functionality in suppressing gas-phase flammability and enhancing condensed-phase electrochemical stability, making it a promising candidate for next-generation, high-safety, high-performance LIB electrolytes.

## 1. Introduction

Modern energy storage has been transformed by the evolution of LIBs, which are now the main component of many portable devices, electric cars, and renewable energy storage systems [1,2]. Despite their widespread adoption, LIBs face major challenges, including safety concerns related to flammability and thermal instability; these are commonly related to the use of LiPF_6_ liquid electrolytes in organic carbonate solvents [3,4]. The high volatility and low thermal stability of these electrolytes normally lead to catastrophic thermal runaway events under abusive conditions, such as overcharging, short circuits, or mechanical damage [5,6]. Furthermore, the decomposition of LiPF_6_ in atmospheric moisture can produce hydrofluoric acid (HF), a highly corrosive byproduct which accelerates the degradation of battery components and compromises the long-term performance [7].

Numerous studies have studied to highlight these risks and emphasized the urgent need for stable and safer electrolyte formulations. For instance, studies have demonstrated that the addition of flame-retardant compounds can significantly reduce the flammability of electrolytes while maintaining their ionic conductivity [8,9,10,11]. In the beginning, studies focused on halogenated flame retardants, but these additives raised environmental and toxicity concerns [12,13,14]. Recent efforts have been moving toward non-halogenated, phosphorus-based additives, which offer a safer and more environmentally friendly approach [15,16]. Additives such as trialkyl phosphates and phosphites have shown promise in enhancing the thermal stability and safety of LIBs [17,18,19,20]. Among the numerous candidates, phosphate-based compounds, due to their high thermal stability and flame-retardant properties, have garnered significant attention. Additives such as ADP, TMP, and TMPi have been explored for their ability to improve both the safety and electrochemical performance of LIBs.

ADP offers several advantages including its inherent flame-retardant properties and less burning potential [21,22]. ADP is an inorganic salt with a straightforward molecular structure comprising ammonium and dihydrogen phosphate ions [23]. Importantly, the decomposition of ADP at elevated temperatures releases non-flammable gases and immediately forms a thermally stable protective layer on electrode surfaces [24]. The formation of a protective layer can inhibit electrolyte decomposition and suppress the growth of the solid electrolyte interphase (SEI) beyond optimal levels, thus contributing to improved cycling stability and capacity retention [25]. Research on the use of ADP in LIBs has demonstrated its effectiveness in reducing the heat release rate and flame propagation of LiPF_6_-based electrolytes without compromising their ionic conductivity [26]. Unlike other flame retardants, ADP has no effect on the electrolyte’s viscosity, which is essential for preserving effective ion transport during the electrochemical process. Furthermore, the long-term electrochemical performance of LIBs is improved by ADP’s capacity to inhibit adverse interactions between the electrolyte and electrode materials. On the other hand, TMP and TMPi are organophosphates which have shown a broader scope of phosphorus-based additives in energy devices. TMP, containing a fully oxidized phosphate group (P=O), is well known for its high oxidation stability, low volatility, and strong flame-retardant capability [27]. TMP’s molecular structure allows to improve electrolyte thermal resistance and suppress combustion without severely affecting ionic conductivity [27]. In contrast, TMPi containing a phosphite group (P–H) in a lower oxidation state, can undergo redox reactions at the electrode–electrolyte interface [28]. This property of TMPi may facilitate the formation of a more stable and conductive SEI/CEI layer, thereby improving cycle stability and Coulombic efficiency [20,29]. By comparing these three structurally distinct phosphorus-based additives (ADP, TMP, TMPi), we aim to systematically evaluate the relationship between chemical structure, thermal stability, flammability suppression, and electrochemical performance in Li-ion battery electrolytes.

This study focuses on evaluating ADP, TMP, and TMPi as promising multifunctional additives for LiPF_6_-based liquid electrolytes, aiming to enhance both thermal and electrochemical stability. Among these, ADP demonstrated the most outstanding performance, and the study aims to elucidate the fundamental mechanisms by which ADP improves thermal stability and cycling performance. Additionally, the effects of ADP on the flammability and electrochemical behavior of LiPF_6_-based electrolytes are thoroughly investigated to determine the most effective additive. From the different characterizations, the fabricated LIBs possess improved stability and performance as ADP strengthens the thermal stability, ionic conductivity, and electrochemical sustainability of the LiPF_6_ electrolyte.

## 2. Materials and Methods

### 2.1. Liquid Electrolyte Preparation

**Figure 1:**
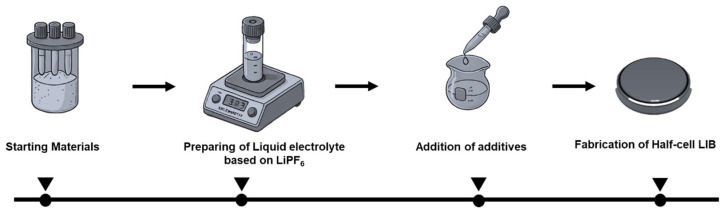
Experimental procedure from materials to device.

For the preparation of electrolytes, a mixture of solvents of ethyl carbonate (EC, 99.9%, Sigma-Aldrich, Burlington, MA, USA), dimethyl carbonate (DMC, 99.9%, Sigma-Aldrich, Burlington, MA, USA), and diethyl carbonate (DEC, 99.0%, Samchun, Seoul, Republic of Korea) were prepared in equal volume ratios (1:1:1 in vol). A total of 1M LiPF_6_ (99.9%, MTI Korea, Seoul, Republic of Korea) was dissolved in prepared solvents and stirred for 2 h followed by sonication for about 4 h at 40 °C. After that, the 1 wt.% phosphorus additives including ADP, TMP (98%, Sigma-Aldrich, Burlington, MA, USA), TMPi (99%, TCI, Tokyo, Japan) were added to the LiPF_6_ solution and the solution was further stirred for 4 h at 40 °C followed by 8 h sonication. The prepared electrolytes were denoted as LiPF_6_, LADP, LTMP, LTMPi for bare LiPF_6_, LiPF_6_ with ADP, TMP and TMPi, respectively, as shown in Figure 1.

### 2.2. Half-Cell Fabrication and Electrochemical Measurement

The half-cell assembly of LIBs was performed in a glove box. In short, the double coated graphite on copper foil (MTI Korea) as the working electrode, lithium foil (MTI Korea) as the counter electrode, and Celgard 2400 (MTI Korea) as the separator were placed layer by layer in a CR2032 stainless steel coin cell. The prepared electrolyte was applied to the electrodes and then sealed under 800–1000 psi pressure. The diameter of electrodes was estimated to 12 mm. Electrochemical measurements, including EIS and C-V, were conducted using a Versa STAT 4 analyzer (AMETEK, Berwyn, PA, USA). EIS was measured across 10 mHz to 100 kHz with a 10 mV RMS AC voltage. Single C-V scans were performed in the 0–6 V range at various scan rates, while multiple C-V scans were conducted at 0.1 mV·s^−1^ and 10 mV·s^−1^ in a range of 0–3 V, and 0–6 V, respectively. The galvanostatic charge–discharge (GCD) was measured conducted by a CR2032 half-cell and carried out on a WonATech WMPG1000 (WonATech, Seoul, Republic of Korea) battery testing system from 1.5 to 3 V at 0.5 C of scan rate.

### 2.3. Characterizations

The liquid electrolytes based on LiPF_6_ with and without additives were characterized by thermal gravimetric analyses (TGA, TGA Q5, TA Instrument, New Castle, DE, USA), differential scanning calorimetry (DSC), and SET. The decomposition point (Td) was determined by TGA test; 10 mg of sample was placed on the platinum pan which was covered by a furnace under nitrogen gas flow. The temperature was increased from room temperature to 800 °C at a rate of 5 °C·min^−1^. DSC analysis was performed by DSC Q20 of WATERS, TA instrument, New Castle, DE, USA. Approximately 10 mg of the sample was sealed in an aluminum pan heated up to 300 °C and kept for 5 min, then by natural cooling to room temperature. A heating rate of 5 °C·min^−1^ was used. The melting point (T_m_) of the sample was determined by the onsets of the endothermic phase transitions. SET measurement was performed in the room temperature.

## 3. Results and Discussion

### 3.1. Thermal Properties

**Figure 2:**
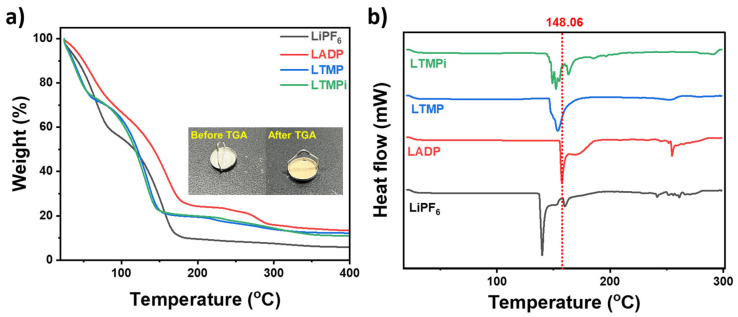
(**a**) TGA and (**b**) DSC of LiPF_6_ (black) with and without ADP (red), TMP (blue), TMPi (green); inset shows the sample ADP before and product after the TGA test.

The TGA analysis results (Figure 2a and Table 1) provide a deeper understanding of the thermal decomposition behavior of LiPF_6_-based electrolytes with and without phosphate additives. In bare LiPF_6_, three distinct decomposition temperatures are observed at 54.89 °C, 138.78 °C, and 221 °C, corresponding to the decomposition of the solvent system (Td_1_), followed by the degradation of minor intermediate phases (Td_2_), and finally the decomposition of LiPF_6_ itself (Td_3_), respectively. This profile underscores the thermal instability of the LiPF_6_ electrolyte, which poses significant safety risks. However, the incorporation of phosphate additives markedly altered the decomposition profile, not only enhancing the thermal stability but also influencing the individual decomposition steps. For LADP, the decomposition temperatures are shifted to Td_1_ at 61.06 °C, Td_2_ at 160.05 °C, and Td_3_ at 278.60 °C. This increase in Td_1_ indicates that ADP interacts with the solvent system which considerably improves its thermal resistance and delays solvent decomposition. Moreover, the rise in Td_3_ reveals that ADP also enhances the thermal stability of LiPF_6_ itself related to its dual mechanisms of gas-phase suppression and the formation of condensed-phase polyphosphate residues [30,31]. These changes not only act as a flame-retardant barrier but also mitigate the decomposition of LiPF_6_ by stabilizing the thermal environment. Other LTMP and LTMPi exhibit the improvements in thermal stability. Both additives delay the decomposition onset of the solvent system and moderately increase the decomposition temperature of LiPF_6_. However, TMP and TMPi additives are less pronounced than that of ADP additive. These findings highlight the multifaceted role of phosphate-based additives, particularly ADP, in improving the thermal stability of the electrolyte system. The ability of ADP to enhance both Td_1_ and Td_3_ demonstrates its unique advantage in providing comprehensive thermal stabilization, making it a highly promising additive for improving the safety and reliability of LIBs at a high temperature.

The DSC analysis provides crucial insights into the thermal behavior of the LiPF_6_ liquid electrolyte containing ADP, TMP, and TMPi. The results in Figure 2b and Table 1 demonstrate that the bare LiPF_6_ electrolyte exhibits the earliest onset of melting (133.84 °C), indicating lower thermal stability. In contrast, LiPF_6_ electrolytes incorporating ADP show a significant increment in melting temperature (148.06 °C), suggesting the strengthening thermal resilience. This enhancement is attributed to ADP’s ability to form a thermally stable polyphosphate layer, which acts as a barrier to heat transfer and prevents early thermal degradation. Other LTMP and LTMPi electrolytes improve the thermal stability by observing the melting temperatures at 141.21 °C and 142.5 °C, respectively, but these values are lower than the LADP electrolyte. DSC analysis clearly presents the potential of phosphate-based additives to enhance the safety and thermal performance of LiPF_6_-based electrolytes. Among the candidates, the ADP-containing electrolyte has emerged as the most promising, owing to its dual function of suppressing gas-phase radicals and stabilizing the condensed phase.

### 3.2. Flammability Test Results

**Figure 3:**
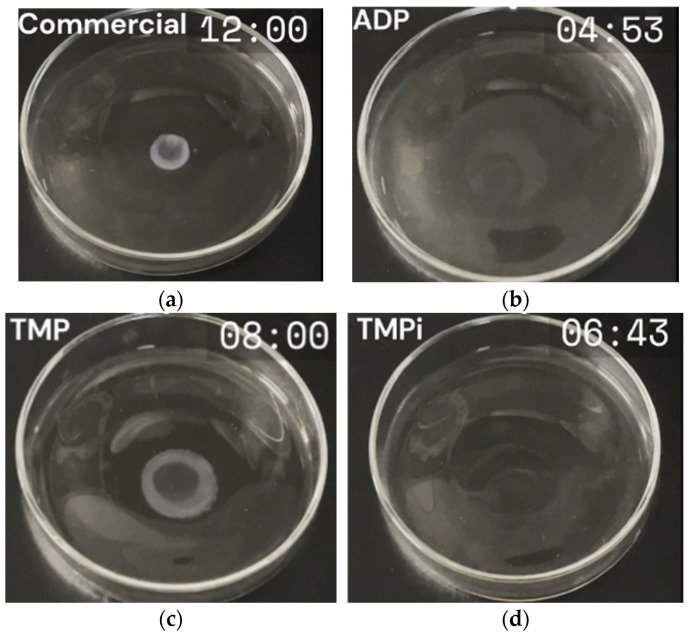
SET results of (**a**) LiPF_6_, (**b**) ADP, (**c**) TMP, (**d**) TMPi.

The flame resistance of the LiPF_6_-based electrolytes with ADP, TMP, and TMPi additives was evaluated through a SET test, as shown in Figure 3. The results reveal a clear improvement in flame suppression when phosphate-based additives are incorporated into the LiPF_6_ electrolyte. The bare LiPF_6_ electrolyte displays the longest combustion duration of 12 min (Figure 3a), indicating high flammability and poor resistance to combustion. In contrast, the LADP, LTMP, and LTMPi electrolytes show a significant reduction in the combustion duration. LiPF_6_ with ADP exhibits the shortest combustion duration of 04 min 53 s (Figure 3b), highlighting its superior flame-retardant effect. This reduction can be attributed to ADP’s ability to release non-flammable gases, such as ammonia and water vapor, which dilute flammable components and interrupt combustion pathways [32,33]. Additionally, ADP likely promotes the formation of a condensed-phase polyphosphate barrier, further inhibiting flame propagation. LTMP and LTMPi electrolytes also demonstrate reduced combustion time, as displayed in Figure 3c (08 min) and Figure 3d (06 min 43 s), respectively. Thus, ADP as an additive is the most effective flame-retardant counterpart, significantly reducing combustion time and enhancing the safety of LiPF_6_-based electrolytes. These findings align with TGA and DSC results, deducing the excellent thermal stability of the LiPF_6_ electrolyte with ADP as an additive.

### 3.3. Electrochemical Measurements

**Figure 4:**
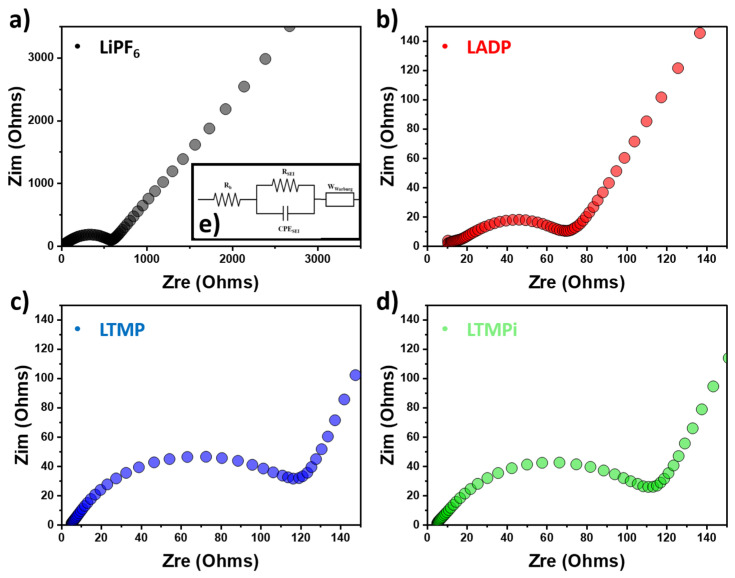
Nyquist plots of (**a**) LiPF_6_; (**b**) LADP; (**c**) LTMP; (**d**) LTMPi for like-fresh cells; and (**e**) an equivalent circuit model of a Li battery half-cell.

As shown in Figure 4 and Table 2, the EIS spectra provide detailed insights into the electrochemical properties of electrolytes containing various phosphate-additives. The bare LiPF_6_ electrolyte presents the largest impedance value, as evidenced by the large semicircle in the Nyquist plot (Figure 4a–d), which suggests the poor interfacial ion transport and high resistance at the electrode–electrolyte interface. Remarkably, the LADP electrolyte (red) shows the lowest impedance among all samples, demonstrating the superior efficiency in improving ion transport and reducing interfacial resistance, whereas LTMP (blue) and LTMPi (green) electrolytes also express the reduced interfacial resistance, but higher than the LADP electrolyte. ADP additive in LiPF_6_ with lower interfacial resistances might enhance the interface behavior at the SEI layer and ionic conductivity.

**Figure 5:**
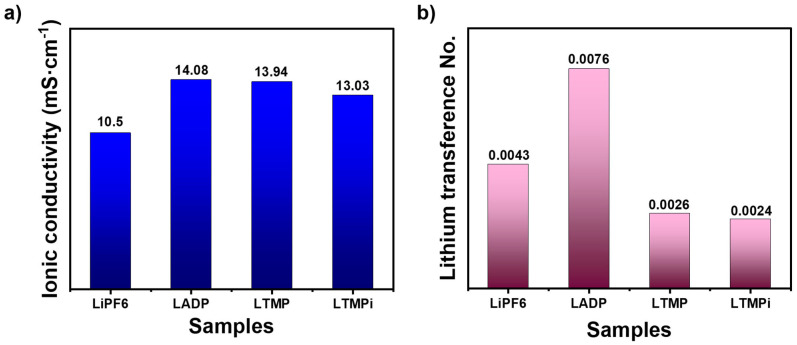
Calculated (**a**) ionic conductivity; and (**b**) lithium transference number from the Nyquist plot.

The ionic conductivity was determined from the results of the EIS measurements, as illustrated in Figure 4b. The bulk resistance (R_b_) was extracted from the low-frequency intercept on the real axis of the Nyquist plot. Ionic conductivity is a critical parameter, as the internal resistance of a battery cell largely arises from the electrical resistivity of the electrolyte solution [34]. The frequency-dependent ionic conductivity of the electrolytes was calculated using the following Equation (1):

(1) $$\sigma =\frac{t}{A\cdot R_{b}}$$

where t = thickness of the electrolyte layer (0.13 in cm), and A = effective contact area (1.2 cm^2^). Figure 5a presents the ionic conductivity results of the prepared electrolytes, revealing the significant differences in conductivity values among the samples. The estimated conductivity values are as follows: LiPF_6_ (10.50 mS·cm^−1^), LADP (14.07 mS·cm^−1^), LTMP (13.94 mS·cm^−1^), and LTMPi (13.03 mS·cm^−1^). The lowest ionic conductivity by bare LiPF_6_ is likely due to stronger ion–ion interactions within the electrolyte, leading to lower ion dissociation and reduced mobility. It is seen that the addition of phosphate-based additives (ADP, TMP, and TMPi) is crucial to enhance the ionic conductivity of the LiPF_6_ electrolyte system. The LADP electrolyte shows the highest ionic conductivity of 14.08 mS·cm^−1^, reflecting its superior ability to facilitate ion transport within the electrolyte. This improvement may be attributed to ADP’s dual role in stabilizing the electrolyte structure and reducing ion association, thereby promoting the dissociation of LiPF_6_ and enhancing ion mobility [35]. The smaller effect of TMP and TMPi additives may be related to their molecular structures and less effective stabilization of the electrolyte or weaker interactions with LiPF_6_. Thus, ADP in LiPF_6_ stands out as the most effective additive, suggesting its potential to enhance the ionic conductivity and electrochemical performance of LIB electrolytes.

Additionally, the lithium-ion transference number ($t_{{\mathrm{Li}}^{+}}^{\mathrm{current}}$) of the liquid electrolyte was evaluated using a refined and elegant one-step very-low-frequency electrochemical impedance spectroscopy (VLF-EIS) method [36]. In the EIS spectra, the low-frequency region primarily reflects ion migration across the electrolyte-soaked separator matrix, which includes contributions from the bulk resistance (R_b_ or R_bulk_)—arising from both ionic transport and interfacial contact resistance. At higher and intermediate frequencies, the impedance response is typically attributed to ion diffusion and the formation of the SEI between lithium metal and the electrolyte, respectively. This region is commonly modeled by a charge transfer resistance (R_ct_) and SEI layer resistance, both contributing to the interfacial impedance at the lithium/electrolyte interface [37]. Unlike the approach proposed by Wohde and Roling, where SEI and charge transfer processes are modeled independently, this study adopts a simplified equivalent circuit: a single resistor–constant phase element (R_in_-CPE) pair to collectively represent both SEI and charge transfer contributions. This simplification is justified due to the similar time constants of these processes, making them difficult to decouple in traditional circuit fitting procedures. Furthermore, the presence of a diffusion-controlled layer within the bulk electrolyte is captured by incorporating a Warburg short (W_s_) element, accounting for semi-infinite linear diffusion. In this model, R_diffusion_ represents the ionic diffusion resistance, τ is the characteristic time constant for the steady-state concentration gradient to develop, and α is the dispersion exponent that reflects non-ideal behavior [36].

(2) $$Z_{W_{s}}={\;R}_{\mathrm{diffusion}}\;.\;\frac{\tan h\left[{\left(j\omega \tau \right)}^{\alpha }\right]}{{\left(j\omega \tau \right)}^{\alpha }}$$

The extracted values from EIS plot are tabulated in Table 2.

Using the values of R_bulk_ and R_diffusion_ provided in Table 2, the lithium-ion transference number ($t_{{\mathrm{Li}}^{+}}^{\mathrm{current}}$) can be estimated according to Equation (3) as follows:

(3) $$t_{{\mathrm{Li}}^{+}}^{\mathrm{current}}=\;\frac{R_{\mathrm{bulk}}}{R_{\mathrm{bulk}\;}+{\;R}_{\mathrm{diffusion}}}$$

The resulting transference numbers are plotted in Figure 5b. The $t_{{\mathrm{Li}}^{+}}^{\mathrm{current}}$ analysis provided key insights into the effect of different phosphate-based additives on lithium-ion mobility in LiPF_6_-based electrolytes. The baseline $t_{{\mathrm{Li}}^{+}}^{\mathrm{current}}$ of the pristine LiPF_6_ electrolyte was found to be 0.0047. When ammonium dihydrogen phosphate (ADP) was introduced, $t_{{\mathrm{Li}}^{+}}^{\mathrm{current}}$ increased to 0.0076, suggesting that ADP can moderately suppress PF_6_^−^ anion migration and slightly improve selective Li^+^ transport. This may stem from possible ionic interactions or weak coordination between the ADP and anions in the electrolyte matrix. In contrast, the use of TMP and TMPi resulted in lower $t_{{\mathrm{Li}}^{+}}^{\mathrm{current}}$ values of 0.0026 and 0.0024, respectively. These reductions imply that both TMP and TMPi, despite their flame-retardant properties, might introduce solvation environments or molecular interactions that do not favor Li^+^ conduction—possibly through stronger coordination with anions or through viscosity increases that hinder ionic transport. Overall, while ADP does not yield the highest conductivity or transference number, it offers a balance between ionic mobility and thermal stability improvements compared to TMP and TMPi under the tested conditions.

**Figure 6:**
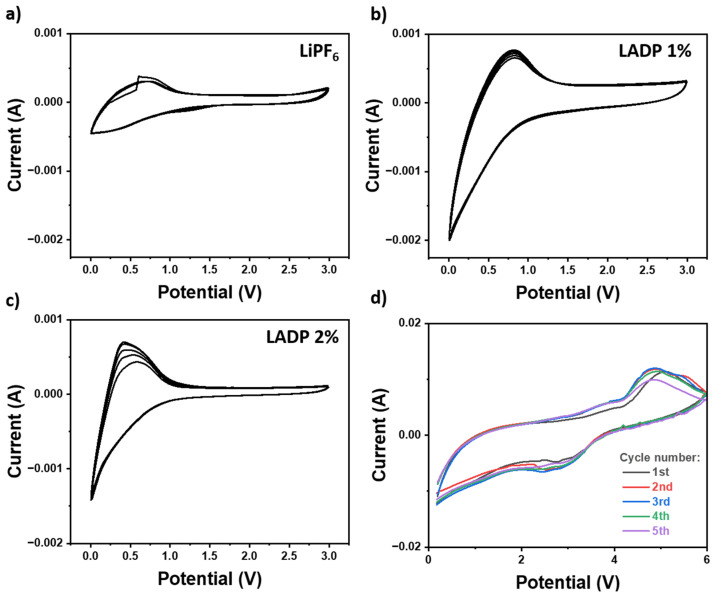
Multicycle C-V curves in the range of 0 to 3 V at a scan rate of 0.1 mV: (**a**) pristine LiPF_6_ electrolyte; (**b**) 1 wt.% ADP in LiPF_6_; (**c**) 2 wt.% ADP in LiPF_6_; (**d**) multicycle C-V curves in the range of 0 to 6 V at a scan rate of 10 mV·s^−1^ of 1 wt.% ADP in LiPF_6_.

The influence of the ADP additive in the LiPF_6_ electrolyte has been studied by measuring the multicycles C-V using a potential window from 0 to 3 V, as displayed in Figure 6. Multicycle C-V analysis is generally used to investigate the process of the intercalation–deintercalation of lithium-ion in the graphite anode after the addition of the ADP additive in the LiPF_6_ electrolyte. Figure 6a shows the typical C-V behavior of the graphite anode with the LiPF_6_ electrolyte as it displays a defined redox peak at ~0.7 V (cathodic) and ~1.1 V (anodic) emerge, which correspond to the reversible lithiation and delithiation processes of graphite (Li^+^ + C ⇄ Li_x_C_6_) [38]. After the addition of ADP (1 wt.% and 2 wt.%) in LiPF_6_ electrolytes, the redox area in C-V markedly improves, as seen in Figure 6b,c. Importantly, after 5 C-V cycles from 0 to 3 V, 1% ADP in LiPF_6_-based electrolyte demonstrates the excellent repeatability or symmetrical with negligible difference from 1st cycle to 5th cycle, which explains that the incorporation of ADP considerably strengthens the reversibility and electrode/electrolyte interfacial stability of the device. At high ADP additive (2 wt.%), the redox area in C-V is further decreased, having the marginal gap between the 1st cycle to the 5th cycle. The higher amount of ADP in the LiPF_6_ electrolyte might increase the viscosity, hinder the electrode wetting, and disturb the uniformity of SEI. Additionally, Figure 6d displays the C-V profiles of the 1% LADP over five consecutive cycles in the voltage range from 0 to 6 V at 10 mV·s^−1^. As compared with C-V at a low potential window and scan rate, a broad oxidation peak appears around 4.9 V in the 1st cycle, which might originate from the initial oxidative decomposition of the carbonate solvents (EC, DMC, DEC) and the breakdown of LiPF_6_ into LiF and PF_5_ [31,39,40,41]. These reactions are irreversible and result in the formation of the initial cathode electrolyte interphase (CEI), which is critical in determining the interfacial stability of high-voltage cathodes [41]. After the 1st cycle, the oxidation region bifurcates into two distinct peaks located approximately at 4.5 V and 5.2 V, respectively, as seen in the 2nd and 3rd cycles, which suggests the further oxidation of remaining carbonate solvents and residual surface species [42]. Notably, in 4th and 5th cycles, the oxidation peak at ~5.2 V disappears, having a stable peak at ~4.5 V. This transition happens due to the formation of a dense and stable CEI layer via passivation at high voltage. This stabilization effect effectively suppresses unwanted side reactions at a high voltage, leading its electrochemical reversibility and longer cycle life. Thus, a 1% LADP formulation can be promised to achieve a substantial reduction in interfacial resistance and to suppress the high-voltage oxidative current.

**Figure 7:**
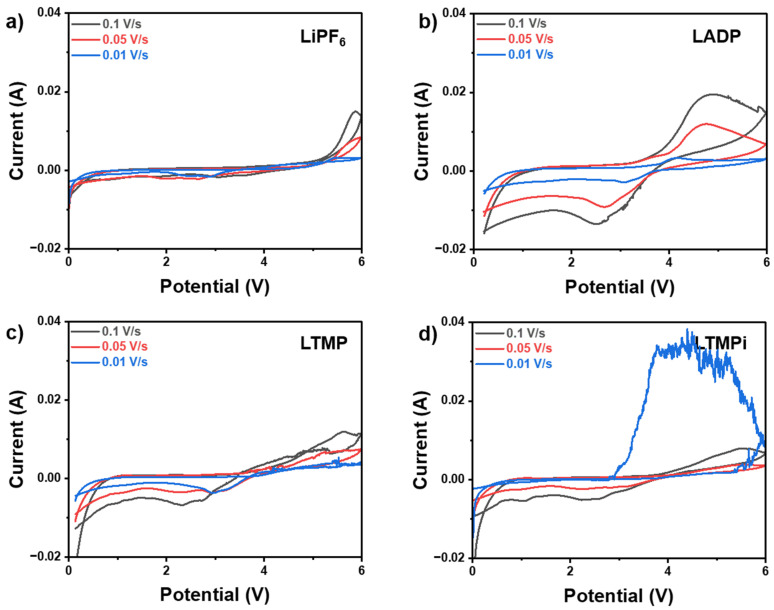
Electrochemical behavior of (**a**) pristine LiPF_6_, (**b**) LADP, (**c**) LTMP, (**d**) LTMPi measured by C-V in the voltage range of 0–6 V at various scan rates (0.01–0.1 V·s^−1^).

The C-V results using a high potential window (0–6 V) with a different scan rate (0.01–0.1 V·s^−1^) are shown in Figure 7, which provide valuable information on the electrochemical stability and redox behavior of LiPF_6_ and LiPF_6_-based electrolytes with phosphate-additives (1 wt.% ADP, 1 wt.% TMP, and 1 wt.% TMPi). The bare LiPF_6_ electrolyte (Figure 7a) exhibits a relatively wide electrochemical stability window with the oxidation onset observed around 4.5–5.0 V. However, the sharp rise in current beyond this range highlights its susceptibility to oxidative degradation at a high potential. LADP electrolytes (Figure 7b) display enhanced oxidative stability with the oxidation onset near 4.5 V and a more symmetric C-V profile, which is indicative of improved redox reversibility. These enhancements can be attributed to ADP’s ability to scavenge radicals and form a thermally stable protective polyphosphate layer, which suppresses side reactions and improves overall electrochemical stability. As compared to LiPF_6_, LTMP (Figure 7c) and LTMPi (Figure 7d) electrolytes also demonstrate improved oxidative stability, although their performances are inferior to LADP. In LTMP and LTMPi electrolytes, the sharper current increase at higher potentials, suggesting a less effective stabilization mechanism and undergoing the side reactions or decomposition under high potentials. These observations deduce the instability of devices during the operation of a large potential window. Therefore, ADP in LiPF_6_ exhibits a more gradual and stable current rise, reflecting better ion transport and reduced diffusion limitations which might show the highest capacity of LIBs.

**Figure 8:**
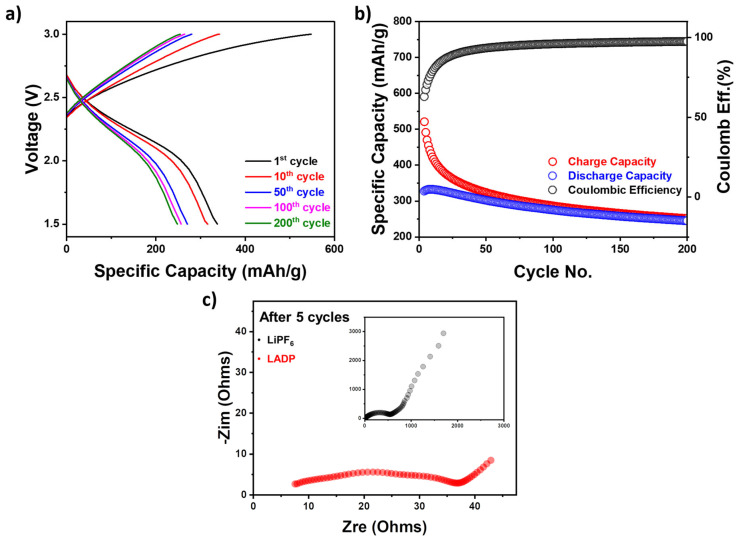
(**a**) Galvanostatic capacity-discharge (GCD) profiles of the LADP sample measured in the voltage range of 1.5–3 V at a scan rate of 0.5 C; (**b**) cycling performances of the LADP sample over 200 cycles; and (**c**) the Nyquist plot of LiPF_6_ (black) and LADP (red) electrolytes after 5 cycles.

Figure 8 collectively elucidates the electrochemical behavior and stability enhancement effects imparted by the incorporation of ADP into LiPF_6_-based electrolytes. The GCD profile in Figure 8a of the 1% LADP electrolyte displays a symmetric voltage-time behavior with a wide potential window up to 1.5–3.0 V, indicating electrochemical stability under prolonged operation. During the initial cycle, a relatively high charge capacity (~506 mAh·g^−1^) is observed, which can be attributed to irreversible processes such as electrolyte decomposition, thicker SEI formation, and surface side reactions over electrolyte/electrode materials [43]. The formation of a thicker SEI layer might hinder the proper Li^+^ ion transport and elevate the resistance, resulting in irreversible capacity loss [44]. Moreover, the unwanted side reactions during the initial charging might ingest the active Li and LiPF_6_ electrolyte, which initially deliver high-charging capacity. However, from the 10th cycle onward, the voltage profiles become increasingly stable, and the polarization is notably reduced. The shape consistency of the charge–discharge curves up to the 200th cycle reflects the formation of a robust and protective interphase, likely promoted by the phosphate species from ADP. In support of this, Figure 8b illustrates the long-term cycling performance and Coulombic efficiency of the cell. The initial charge capacity reaches ~506 mAh·g^−1^ with an initial discharge capacity of ~320 mAh·g^−1^, corresponding to a first cycle Coulombic efficiency of ~64%. During initial cycles, the irreversible reactions occur due to the reduction of carbonate solvents (EC, DEC, DMC) and the decomposition of LiPF_6_ to generate a thick SEI layer containing both organic species [(CH_2_OCO_2_Li)_2_] and inorganic species (Li_2_CO_3_, LiF), tending to permanent Li loss [45,46,47]. In addition, the use of ADP as an additive might partially decompose to form inorganic species such as Li_3_PO_4_, polyphosphates, and P–F-containing species, which are insoluble, electronically insulating, and remain fixed on the electrode surface, that are permanently incorporated into the interphase layers [47,48]. This implies high reversibility of the redox processes and minimal ongoing unwanted side reactions, increasing the Li^+^ ion transport and capacity [49]. However, the subsequent cycles show a rapid increase in Coulombic efficiency, it stabilizes ~98% and maintains up to 200 cycles. It might be attributed to the incomplete suppression of unwanted reactions during Li deposition/stripping [48]. Although the ADP-containing electrolyte forms a stable inorganic-rich interphase that mitigates continuous electrolyte decomposition, the SEI is not perfectly passivating. However, a small portion of Li^+^ is still irreversibly consumed via side reactions between freshly exposed Li metal and electrolyte components [49], and afterward, the generated phosphate species might stabilize the SEI and recover Li loss, thus further elevating the Coulombic efficiency [50]. Thus, these factors collectively maintain the Coulombic efficiency ~98% up to 200 cycles after initial loss. As seen in Table 3, the Coulombic efficiency in Graphite|Li cell shows the higher value as compared to the reported Li-Li symmetric cell [50].

As seen in Figure 8b, the gradual discharge capacity fading is observed during the first 50 cycles and retains a stable discharge capacity of ~220 mAh·g^−1^ up to 200 cycles. After initial capacity fade, the discharge capacity and Coulombic efficiency of the device are highly stable up to 200 cycles, which is related to the flame-retardant and interfacial-stabilizing effects of the ADP additive. During the stabilization, ADP might form a condensed-phase protective phosphate layer and suppress electrolyte decomposition and gas evolution [27]. Overall, the results highlight the potential of ADP as a dual-function additive that enhances both the safety and electrochemical reliability of LiPF_6_-based electrolytes in lithium-ion batteries, balancing performance and thermal stability for advanced energy storage applications.

As shown in Figure 8c, EIS was employed to evaluate the interfacial resistance and charge transfer characteristics of the lithium-ion cell using a 1% LADP-based electrolyte. As illustrated in the Nyquist plot, the impedance spectrum of the 1% LADP-based electrolyte is characterized by a depressed semicircle in the high-to-medium frequency region, followed by an inclined linear tail in the low-frequency region. In LIBs, the semicircle corresponds to the R_ct_ at the electrode/electrolyte interface, and the linear portion relates to the diffusion of Li^+^ ions in the electrode material (Warburg impedance) [51]. As compared to the LiPF_6_ electrolyte (black), the 1% LADP (red) depicts a smaller semicircle diameter, indicating a much lower R_ct_, which translates into enhanced charge transfer kinetics. Herein, the ADP additive likely facilitates the formation of phosphate-rich SEI components, which have been reported to possess better chemical stability and lower impedance [52]. Furthermore, the occurrence of the linear shape at the low-frequency region by the 1% LADP-based electrolyte is steeper as compared to the LiPF_6_ electrolyte, indicating improved Li^+^ diffusion properties through the electrolyte and the electrode matrix. This behavior is consistent with the observed high ionic conductivity and enhanced electrochemical performance of the LADP-based system in GCD and cycling measurements. The findings suggest that the incorporation of the ADP additive not only enhances thermal stability and flame retardancy but also actively promotes faster interfacial lithium-ion transport, thereby improving the overall rate capability and cycle life of the cell [53].

### 3.4. ADP’s Role in Enhancing LIB Safety and Electrochemical Properties

**Figure 9:**
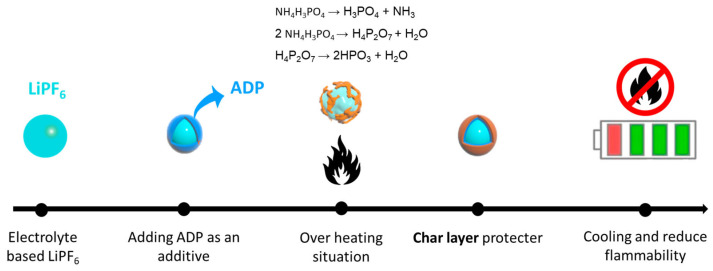
Flammable retardant of ADP in LIBs based on LiPF_6_.

As illustrated in Figure 9, ADP as an additive in the LiPF_6_ electrolyte plays a pivotal role in improving the safety of LIBs by addressing critical challenges such as flammability and thermal runaway through a combination of chemical and physical mechanisms. During thermal decomposition, ADP releases non-flammable gases, including ammonia (NH_3_) and phosphoric acid derivatives, which serve as radical scavengers. Radical scavengers effectively suppress flammability by interrupting chain reactions involving reactive radical species (e.g., H, OH) generated during electrolyte decomposition, thereby enhancing the thermal stability and safety of lithium-ion batteries [54,55]. In addition, ADP facilitates the formation of a thermally stable polyphosphate layer on the surface of battery components, particularly the electrodes [56]. This condensed-phase protective layer acts as a physical barrier, isolating the flammable electrolyte from heat and oxygen while simultaneously stabilizing the SEI. This barrier not only reduces the risk of combustion but also enhances the long-term stability of the electrolyte and battery system. Another critical feature of ADP is its endothermic decomposition process, which absorbs heat from the surrounding environment.

This absorption lowers the local temperature, delaying the onset of thermal runaway and further contributing to thermal stabilization. Furthermore, the release of non-flammable gases like NH_3_ and H_2_O dilutes the concentration of combustible materials within the battery, adding an additional layer of protection against ignition and flame propagation. It is supported by the observation in TGA and DSC results. The TGA results (Figure 2b) provide compelling evidence of ADP’s flame-retardant mechanism in which the post TGA sample appeared as gray-brown residue as compared to before TGA. The conversion of the sample’s color is indicative of a polyphosphate-rich layer formed during thermal decomposition which aligns with the proposed flame-retardant mechanism, wherein ADP releases non-flammable gases while promoting the formation of this thermally stable condensed-phase layer. In support, this residue acts as a critical barrier, shielding the electrolyte components, particularly LiPF_6_, from further decomposition, resulting in improving the thermal stability and combustion duration time of LiPF_6_. In summary, ADP’s synergistic effects in both the gas phase (via radical scavenging and gas dilution) and the condensed phase (via the formation of protective polyphosphate layers) significantly enhance the thermal stability and safety of LiPF_6_-based electrolytes. These properties make ADP an excellent candidate for use as a flame-retardant additive in LIBs, offering improved safety without compromising electrochemical performance.

## 4. Conclusions

In this study, the effects of phosphate-based additives (ADP, TMP, TMPi) on the thermal and electrochemical performance of LiPF_6_-based electrolytes were systematically explored. The incorporation of these additives significantly enhanced the ionic conductivity, thermal stability, and flame-retardant properties of the electrolytes. Among them, the ADP-containing electrolyte exhibited the most pronounced improvements, attributed to its ability to form thermally stable polyphosphate networks that suppress flammable gas evolution. Electrochemical analyses, including C-V and EIS, revealed that ADP not only improved ionic conductivity (reaching 14.07 mS·cm^−1^) but also reduced interfacial resistance at the SEI after prolonged cycling. Furthermore, the $t_{{\mathrm{Li}}^{+}}^{\mathrm{current}}$ of the ADP-based electrolyte was recorded at 0.0076, surpassing that of the pristine LiPF_6_ electrolyte (0.0047), indicating improved Li^+^ mobility due to ADP’s ionic interaction environment. GCD and multiple charge–discharge tests demonstrated enhanced long-term cycling stability and capacity retention, especially in the sample with LADP, which balanced interfacial kinetics and electrolyte stability. The flame-retardant mechanism of ADP was further confirmed through SET tests and thermal decomposition studies, showing its role in forming a condensed-phase protective barrier that limits exothermic reactions and suppresses thermal runaway. Collectively, these results establish ADP as the most effective multifunctional additive among the studied phosphates, offering dual-phase stabilization, improved transport properties, and enhanced thermal safety. Thus, the incorporation of the ADP additive into LiPF_6_-based electrolytes presents a promising strategy for developing safer and high-performance lithium-ion batteries, potentially enabling their broader deployment in demanding energy storage systems.

## Figures and Tables

**Table 1:** Specific points of samples from TGA and DSC results.

Samples	Td_1_ (°C)	Td_2_ (°C)	Td_3_ (°C)
LiPF_6_	54.89	138.78	221.00
LADP	61.06	160.05	278.60
LTMP	40.71	131.54	254.56
LTMPi	40.25	131.78	255.10

**Table 2:** The particle resistance values from the Nyquist plot.

Sample	R_bulk_ or R_b_ (Ω)	R_in_ (Ω)	R_diffusion_ (Ω)
LiPF_6_	10.39	568.13	2406.49
LADP	7.75	61.06	1005.94
LTMP	7.82	108.33	2988.98
LTMPi	8.37	102.28	3487.46

**Table 3:** A comparison of electrochemical performance of our results and the reported Li-Li symmetric cell.

Sample	Electrolyte	Coulombic Efficiency
Graphite|Li cell (our work)	1 M LiFP_6_-EC/DEC/DMC 1/1/1 (*v*/*v*)	≥98%
Li|Li symmetric cell [50]	1.1 M LiPF_6_-FEC/EMC 1/19 (*v*/*v*)	~62.5%
	1.1 M LiPF_6_-FEC/EMC 1/14 (*v*/*v*)	~78%
	1.1 M LiPF_6_-FEC/EMC 1/9 (*v*/*v*)	~88%
	1.1 M LiPF_6_-FEC/EMC 1/6 (*v*/*v*)	~92%
	1.1 M LiPF_6_-FEC/EMC 1/4 (*v*/*v*)	~98%
	1.1 M LiPF_6_-FEC/EMC 3/7 (*v*/*v*)	~97%

Fluoroethylene carbonate (FEC), ethyl methyl carbonate (EMC).

## Data Availability

Data is contained within the article.
